# Missingness-aware machine learning using routine laboratory data for distinguishing hepatitis from cirrhosis

**DOI:** 10.3389/fgene.2026.1936507

**Published:** 2026-09-22

**Authors:** Qin Yang, Xuerui Hu, Shihong Yu, Tingjun Zhang, Qirong Zhu, Chengxing Yin, Zhihui Ren, Jiayi Feng, Changjie Xue, Juanjuan Kang, Fengjun Liu, Qiang Tang, Xi Yong

**Affiliations:** 1 Department of Infectious Diseases, Affiliated Hospital of North Sichuan Medical College, Nanchong, China; 2 Department of Endocrinology, Affiliated Hospital of North Sichuan Medical College, Nanchong, China; 3 Department of Clinical Medicine, Affiliated Hospital of North Sichuan Medical College, Nanchong, China; 4 Innovative Institute of Chinese Medicine and Pharmacy, Chengdu University of Traditional Chinese Medicine, Chengdu, China; 5 Key Laboratory of Non-Coding RNA and Drug Discovery at Chengdu Medical College of Sichuan Province, School of Basic Medical Sciences, Chengdu Medical College, Chengdu, China; 6 Department of Vascular Surgery, Affiliated Hospital of North Sichuan Medical College, Nanchong, China; 7 Sichuan Branch of National Clinical Research Center for Digestive Diseases, Nanchong, China

**Keywords:** blood routine, hepatitis, liver cirrhosis, machine learning, missingness pattern

## Abstract

**Introduction:**

Early identification of cirrhosis among patients with hepatitis is important for timely risk stratification and clinical management, yet conventional non-invasive scores such as the fibrosis-4 index (FIB-4) and the aspartate aminotransferase-to-platelet ratio index (APRI) may provide limited discrimination. In clinical datasets, missingness patterns may also carry predictive information, but their value for cirrhosis identification remains insufficiently investigated.

**Methods:**

We included 672 individuals, comprising 334 patients with hepatitis and 338 patients with cirrhosis, and randomly divided them into training and testing sets at in 8:2 ratio. FIB-4, APRI and multiple machine-learning models were developed using routinely available laboratory parameters; missing values were handled by median imputation, and binary missingness indicators were incorporated into selected models.

**Results:**

Machine-learning models showed better discriminatory performance than conventional scores. The Extra Trees model incorporating missingness indicators (ET + indicator) achieved the highest AUC (0.841), whereas the support vector machine model incorporating missingness indicators (SVM + indicator) achieved the highest AUPRC (0.813). By contrast, FIB-4 and APRI had AUC values of 0.537 and 0.519, respectively. Incorporating missingness indicators further improved the performance of ET, SVM and HGB models, and reducing the ET + indicator threshold from 0.50 to 0.42 increased sensitivity from 0.765 to 0.882.

**Discussion:**

Missingness-aware machine-learning models based on routine laboratory parameters may improve hepatitis-to-cirrhosis discrimination and support risk stratification in real-world screening settings.

## 1 Introduction

Chronic liver disease is a major global cause of morbidity and mortality, and cirrhosis represents a critical stage in the progression of various chronic liver disorders (Devarbhavi et al., 2023; Huang et al., 2023). In patients with chronic hepatitis, the development of cirrhosis indicates advanced structural and functional liver deterioration. It is also associated with increased risks of portal hypertension, hepatic decompensation and hepatocellular carcinoma (Gines et al., 2021). Timely identification of cirrhosis in patients with hepatitis is therefore important for surveillance, risk stratification, monitoring, intervention planning and prognostic management (Gines et al., 2021; Singal et al., 2022).

Current assessment of cirrhosis relies on imaging, elastography, histopathological examination and non-invasive scoring systems (Ito and Mitchell, 2004; Buti et al., 2024). These approaches provide clinically useful information, but their accessibility, standardization and timing vary across real-world settings. This variability is particularly relevant when rapid initial assessment or large-scale patient stratification is required, making simple and cost-effective screening tools desirable (Gines et al., 2022). The fibrosis-4 index (FIB-4) and the aspartate aminotransferase-to-platelet ratio index (APRI) are widely used because they are simple and clinically accessible (Amernia et al., 2021; Mallet et al., 2019; Santol et al., 2025). However, these scores incorporate only a small number of variables and may not fully capture the multidimensional clinical heterogeneity between hepatitis and cirrhosis. Their discriminatory performance can therefore be limited in populations such as patients with chronic hepatitis B (Xiao et al., 2015; Kim et al., 2016).

Routinely collected laboratory measurements may help address this limitation because they are broadly available, low cost and reproducible. These measurements reflect multiple dimensions of cirrhosis-related pathophysiology, including hepatic synthetic function, coagulation abnormalities, haematological alterations, bilirubin metabolism and renal dysfunction (Lawrence and Steiner, 2017; Mucino-Bermejo et al., 2013; Fierro-Angulo et al., 2024; Castro-Garcia et al., 2014). Because cirrhosis is a systemic clinical phenotype rather than an abnormality of a single biomarker, multivariable modelling of laboratory features may improve discrimination between hepatitis and cirrhosis. With the rapid development of machine learning approaches for biomedical data analysis, data-driven models based on serum biomarkers and clinical variables have increasingly been explored for risk assessment in chronic liver disease, supporting the feasibility of multi-feature predictive modelling in this setting (Zhang et al., 2024; Tang et al., 2026; Kang et al., 2026a).

A key limitation of clinical modelling is that missing data are often treated only as a technical problem. In clinical data, missingness is frequently not completely random but may reflect disease severity, clinical suspicion or healthcare workflows (Groenwold, 2020; Sisk et al., 2021). Conventional modelling strategies commonly remove this potential information through imputation. By contrast, methodological studies suggest that missingness patterns themselves may carry predictive signals (Groenwold, 2020; Sisk et al., 2021; Sisk et al., 2023). In hospitalized patients with liver disease, whether a laboratory test is ordered may reflect both clinical decision-making and underlying disease characteristics. How to preserve and use information encoded in missingness therefore remains an important but insufficiently investigated issue in predictive modelling with real-world liver disease data.

Here, we used routinely collected laboratory data from hospitalized patients to develop and compare conventional clinical scores with multiple machine learning models for distinguishing hepatitis from cirrhosis. We specifically examined whether explicit missingness indicators improve discrimination beyond standard modelling approaches. We also incorporated threshold optimization, calibration analysis and decision curve analysis to evaluate model performance and potential clinical utility in screening-oriented settings. This study addresses the gap between laboratory-based risk stratification and the informative missingness inherent in real-world clinical data.

## 2 Materials and methods

### 2.1 Study design and data source

This single-centre retrospective study used electronic medical records from hospitalized patients at the Affiliated Hospital of North Sichuan Medical College between 1 January 2020 and 31 December 2024. Laboratory measurements collected during the first hospitalisation of each eligible patient were extracted for subsequent analyses. The hepatitis group consisted of patients with discharge diagnoses of acute or chronic hepatitis and without any documented evidence of cirrhosis. The cirrhosis group comprised patients with a discharge diagnosis of liver cirrhosis. Diagnostic support for cirrhosis was required from at least one of the following sources. Typical imaging features were identified on abdominal ultrasonography computed tomography or magnetic resonance imaging. Valid liver stiffness measurement or liver biopsy results were accessible. Patients with contradictory diagnoses ambiguous classification between hepatitis and cirrhosis or incomplete laboratory data from the first hospitalisation were excluded. FIB-4 and APRI were not applied as criteria for enrolment or group assignment. These indices were only calculated for comparative purposes.

### 2.2 Sample selection and preprocessing

To improve data quality and reduce the potential influence of excessive missingness on model training, a two-step filtering strategy was applied. First, patients with fewer than 40 available laboratory measurements were excluded. Second, variables with valid measurements available for fewer than 200 individuals across the entire cohort were removed. After filtering, 672 participants were retained, including 334 patients with hepatitis and 338 patients with cirrhosis. The cohort was divided into training and independent testing sets at an 8:2 ratio with a fixed random seed (random state = 42) to ensure reproducibility. This resulted in 537 patients in the training set and 135 patients in the independent testing set.

Missing rates varied across clinical variables. To enable model training while preserving potentially informative missingness patterns, missing values in continuous variables were imputed using the median value of each variable calculated from the training set. The same imputation parameters were then applied to the testing set to avoid information leakage. For missingness-aware models, binary missingness indicator variables were incorporated after imputation to indicate whether each laboratory measurement was missing.

### 2.3 Clinical variables and conventional scores

The included variables covered haematological parameters, hepatic synthetic and coagulation function markers, liver injury indicators, bilirubin metabolism-related parameters, renal function measurements, hepatitis B virus (HBV) serological markers, tumor biomarkers and demographic characteristics. Detailed variable classifications are provided in Table 1.

**TABLE 1:** Detailed classification of clinical features.

Feature categories	Features
Renal function	SCr, GFR, BUN, UA
Liver injury	AST, ALT, ALP, ADA, a-L-AFU, 5-NT
Bilirubin metabolism	TBIL, DBIL, IBIL
Synthetic/coagulation	CHE, PAB, TP, GLOB, A/G, PT, TT, FIB, INR
Hematology	WBC, NEUT, LY, MONO, Baso, Eos, RBC, Hb, MCHC, HCT, MCH, MCV, PLT, PCT, MPV
Tumor markers	AFP, PIVKA-II
HBV serology	HBeAg, HBeAb, HBcAb
Demographics	Gender, age
Derived scores	FIB4, APRI

In addition to the original clinical variables, two widely used non-invasive clinical scores, the fibrosis-4 index (FIB-4) and the aspartate aminotransferase-to-platelet ratio index (APRI), were calculated as baseline models. FIB-4 and APRI were calculated as follows Equations 1, 2:

$$FIB-4=\frac{age\times AST}{PLT\times \sqrt{AST}}$$ (1)

$$APRI=\frac{\left(AST/40\right)\times 100}{PLT}$$ (2)

The upper limit of normal for AST was set to 40 U/L.

### 2.4 Machine learning algorithms

Several machine learning models were developed using routinely collected laboratory variables. These included a random forest (RF) model and a HistGradientBoosting model without explicit missingness indicators (HGB core). Missingness-aware models were constructed by incorporating missingness indicators and included support vector machine (SVM + indicator), Extra Trees (ET + indicator), XGBoost (XGB + indicator), and LightGBM (LGBM + indicator).

During model training, HGB core was configured with max_depth = 4, learning_rate = 0.05 and max_iter = 220. SVM + indicator was implemented using a radial basis function kernel with *C* = 2.0 and gamma = 0.01. ET + indicator was configured with n_estimators = 800 and the entropy criterion. XGB + indicator and LGBM + indicator was trained using default parameter settings.

### 2.5 Statistical analysis and model evaluation

Continuous variables were summarized as medians with interquartile ranges and compared between groups using the Mann–Whitney U test. Categorical variables were presented as percentages and compared using Fisher’s exact test or the chi-square test, as appropriate.

Cliff’s delta was calculated for continuous variables to quantify the magnitude and direction of distributional differences between groups. Positive values indicated higher overall values in the cirrhosis group, whereas negative values indicated higher values in the hepatitis group. Larger absolute values indicated greater separation between distributions. Correlations among key variables were assessed using Spearman’s rank correlation coefficient.

Model performance was evaluated using accuracy (Acc), Matthews correlation coefficient (MCC), sensitivity (Sn), and specificity (Sp) (Kang et al., 2026b; Tang et al., 2015), calculated as follows Equations 3–6:

$$\text{Acc}=\frac{TP+TN}{TP+TN+FP+FN}$$ (3)

$$\text{Sn}=\frac{TP}{TP+FN}$$ (4)

$$\text{Sp}=\frac{TN}{TN+FP\;}$$ (5)

$$\text{MCC}=\frac{TP\times TN-FP\times FN}{\sqrt{\left(TP+FP\right)\left(TP+FN\right)\left(TN+FP\right)\left(TN+FN\right)}}$$ (6)

Cirrhosis was defined as the positive class, and hepatitis was defined as the negative class. TP and TN represented correctly classified patients with cirrhosis and hepatitis. Accordingly, TP represented patients with cirrhosis correctly classified as cirrhosis, and TN represented patients with hepatitis correctly classified as hepatitis. FP represented patients with hepatitis incorrectly classified as cirrhosis, whereas FN represented patients with cirrhosis incorrectly classified as hepatitis. The area under the receiver operating characteristic curve (AUC) and the area under the precision-recall curve (AUPRC) were also used to assess overall model discrimination.

### 2.6 Ethics statement

This retrospective study was approved by the Medical Ethics Committee of the Affiliated Hospital of North Sichuan Medical College (approval No. 2026ER333-1). The requirement for informed consent was waived by the ethics committee because the study used retrospective de-identified clinical data. The study was conducted in accordance with the Declaration of Helsinki.

## 3 Results

### 3.1 Cohort composition and clinical variables

This study included 672 participants, comprising 334 patients with hepatitis and 338 patients with cirrhosis. After random allocation at an 8:2 ratio, 537 patients were assigned to the training set and 135 to the independent testing set (Figure 1A). The training set included 267 patients with hepatitis and 270 patients with cirrhosis, while the testing set included 67 patients with hepatitis and 68 patients with cirrhosis. Baseline characteristics of the two datasets are summarized in Table 2. Age, sex distribution, disease status, first-hospitalization records, available variable counts and representative laboratory variables were broadly comparable between the training and testing sets, suggesting that the random split did not introduce marked baseline imbalance. The included variables covered multiple clinically relevant laboratory domains (Figure 1B). Haematological parameters and hepatic synthetic/coagulation markers accounted for the largest proportions of variables, at 37.9% and 25.7%, respectively. Liver injury-related markers accounted for 10.6%, renal function indicators for 6.7%, and the remaining variables comprised bilirubin metabolism-related parameters, HBV serological markers, tumor biomarkers, demographic variables, and derived clinical scores.

**FIGURE 1:**
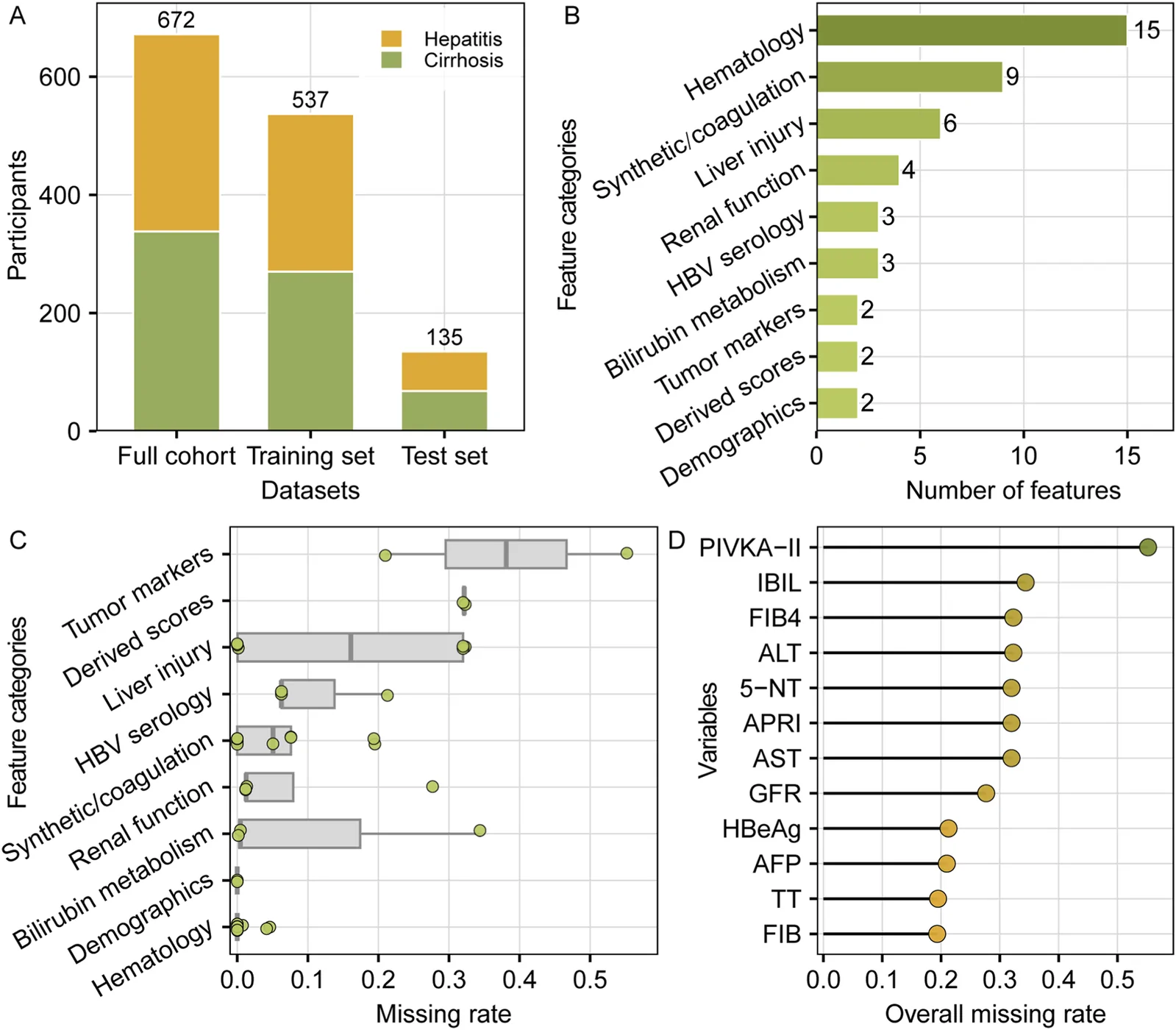
Cohort composition, feature-category structure, and missingness profile of the study dataset. **(A)** Composition of the overall cohort, training set, and independent testing set, including the distribution of patients with hepatitis and cirrhosis. **(B)** Category composition of the included features. **(C)** Distribution of missing rates across different feature categories. **(D)** Ranking of variables with the highest overall missing rates.

**TABLE 2:** Baseline characteristics of the training and testing sets.

Characteristic	Training set (n = 537)	Testing set (n = 135)	P value
Total patients, n	537	135	​
First hospitalization records, n (%)	537 (100.0)	135 (100.0)	​
Hepatitis, n (%)	267 (49.7)	67 (49.6)	0.985
Cirrhosis, n (%)	270 (50.3)	68 (50.4)	​
Male sex, n (%)	412 (76.7)	101 (74.8)	0.641
Age, years, median (IQR)	61.00 (54.00–69.00)	60.00 (54.00–70.50)	0.875
Available variables per patient, median (IQR)	41.00 (39.00–42.00)	42.00 (39.00–43.00)	0.350
SCr, median (IQR)	66.50 (55.35–78.65)	65.40 (53.30–79.90)	0.738
GFR, median (IQR)	101.44 (82.98–121.12)	99.69 (84.45–116.85)	0.707
BUN, median (IQR)	5.32 (4.06–7.51)	5.54 (4.08–7.45)	0.713
AST, median (IQR)	56.62 (36.04–120.83)	59.50 (33.92–115.43)	0.819
ALT, median (IQR)	37.32 (23.46–84.57)	45.60 (24.66–88.60)	0.424
TBIL, median (IQR)	26.83 (17.20–58.49)	26.20 (18.46–72.44)	0.471
CHE, median (IQR)	3400.76 (2414.28–5044.00)	3661.00 (2211.86–6165.00)	0.332
PAB, median (IQR)	76.20 (35.60–147.16)	79.50 (41.00–159.10)	0.336
PT, median (IQR)	15.90 (14.40–18.55)	15.50 (14.00–18.30)	0.317
INR, median (IQR)	1.28 (1.13–1.54)	1.23 (1.10–1.53)	0.290
WBC, median (IQR)	4.93 (3.46–7.05)	4.87 (3.60–7.12)	0.962
Hb, median (IQR)	118.00 (96.00–137.00)	123.00 (97.00–144.00)	0.157
PLT, median (IQR)	79.00 (52.00–135.00)	98.00 (58.00–137.00)	0.118
AFP, median (IQR)	8.90 (2.50–85.07)	8.80 (2.55–95.10)	0.575
PIVKA-II, median (IQR)	31.74 (18.02–183.04)	29.38 (19.10–96.49)	0.764

Abbreviations: IQR, interquartile range; SCr, serum creatinine; GFR, glomerular filtration rate; BUN, blood urea nitrogen; AST, aspartate aminotransferase; ALT, alanine aminotransferase; TBIL, total bilirubin; CHE, cholinesterase; PAB, prealbumin; PT, prothrombin time; INR, international normalized ratio; WBC, white blood cell count; Hb, haemoglobin; PLT, platelet count; AFP, alpha-fetoprotein; PIVKA-II, protein induced by vitamin K absence or antagonist-II.

Missingness varied across feature categories (Figure 1C). Tumor biomarkers and derived scores showed higher missing rates and greater variability. Liver injury-related and renal function indicators also showed varying missingness. By contrast, haematological parameters and demographic variables generally had lower missing rates and more concentrated distributions.

When variables were ranked by individual missing rates, the most frequently missing features were PIVKA-II, indirect bilirubin (IBIL), FIB-4, alanine aminotransferase (ALT), 5′-nucleotidase (5′-NT), APRI, and aspartate aminotransferase (AST) (Figure 1D). Variables with high missingness were mainly concentrated in among tumor-related tests, bilirubin fractionation, liver injury markers and derived scores calculated from primary laboratory variables, rather than being uniformly distributed across all feature categories (Kim et al., 2023; Kwo et al., 2017; Karayiannis et al., 1990).

### 3.2 Cirrhosis was associated with coordinated changes in key clinical indicators

To characterize clinical differences between hepatitis and cirrhosis, six representative variables were compared between groups (Figures 2A–F). Compared with the hepatitis, patients with cirrhosis had lower cholinesterase (CHE), prealbumin (PAB), platelet count (PLT) and haemoglobin (Hb), together with higher prothrombin time (PT) and total bilirubin (TBIL).

**FIGURE 2:**
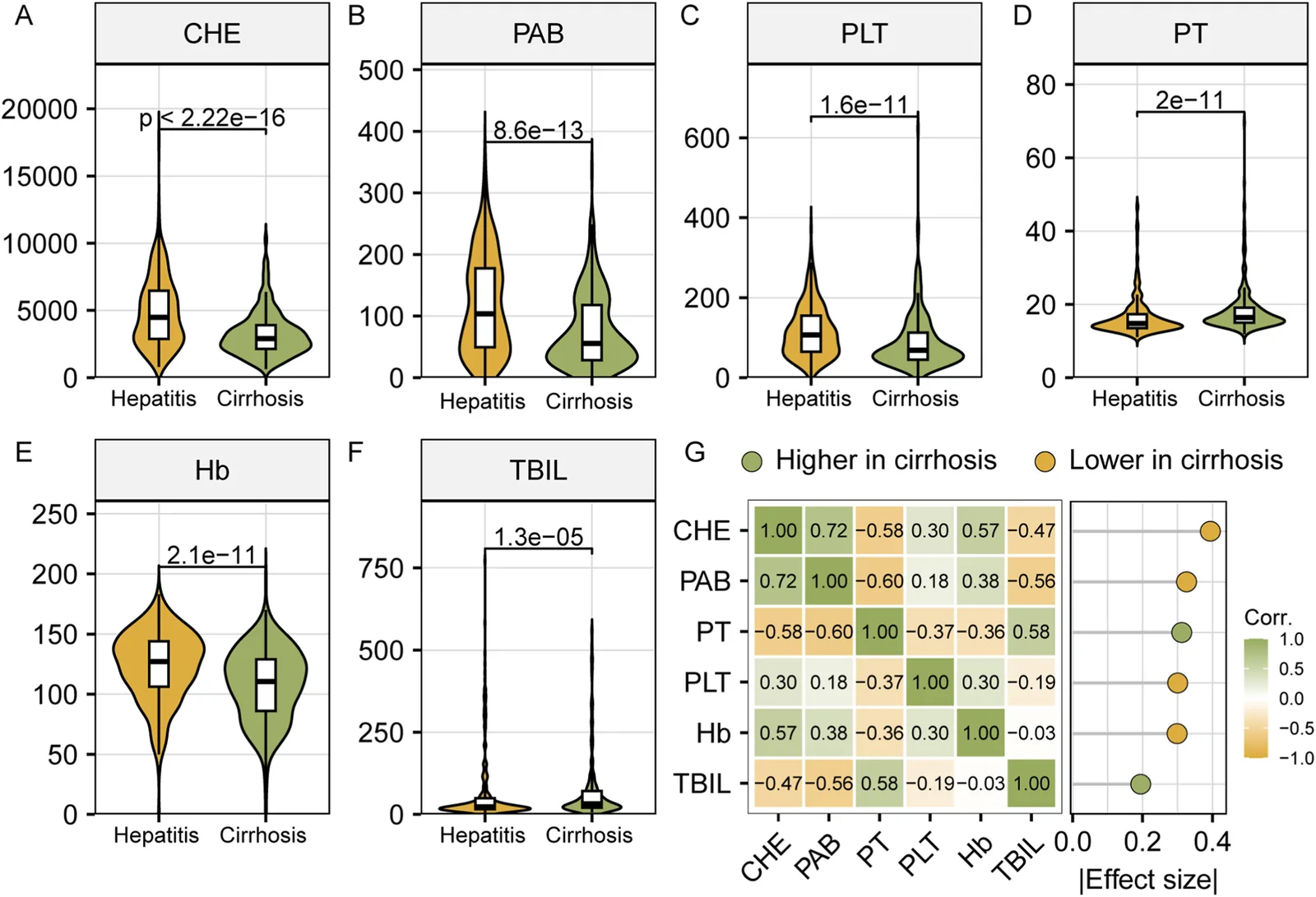
Distributional differences, statistical significance and correlation structure of key clinical markers between hepatitis and cirrhosis. **(A–F)** Distributional differences in CHE, PAB, PLT, PT, Hb, and TBIL between the hepatitis and cirrhosis groups. **(G)** Correlation heatmap of key clinical indicators with corresponding effect-size summaries. Green indicates higher values in the cirrhosis group, whereas orange indicates lower values in the cirrhosis group.

Correlation analysis showed CHE was positively correlated with PAB (Figure 2G). PT was negatively correlated with CHE and PAB and positively correlated with TBIL, suggesting that coagulation impairment covaries with reduced synthetic capacity and increased bilirubin levels (Figure 2G). These findings suggest that different pathological dimensions provide coordinated yet complementary information. Effect-size analysis confirmed higher PT and TBIL levels in the cirrhosis group, whereas CHE, PAB, PLT, and Hb were lower. Collectively, these results suggest that cirrhosis, relative to hepatitis, is characterized not by isolated abnormalities in individual markers but by coordinated multidimensional alterations involving impaired hepatic synthetic function, prolonged coagulation time, haematological decline, and disrupted bilirubin metabolism, with the most pronounced differences observed for CHE, PAB, and PT.

### 3.3 Machine-learning models showed higher discrimination than conventional clinical scores

Model performance was evaluated in the independent testing set (Figure 3). ROC analysis showed that ET + indicator achieved the highest AUC (0.841). SVM + indicator and LGBM + indicator both achieved an AUC of 0.836, followed by XGB + indicator with an AUC of 0.824. HGB core and the RF baseline had AUC values of 0.797 and 0.796, respectively. By contrast, FIB-4 and APRI had lower AUC values of 0.537 and 0.519, respectively. In the precision–recall analysis, SVM + indicator achieved the highest AUPRC of 0.813 (Figures 3A,B).

**FIGURE 3:**
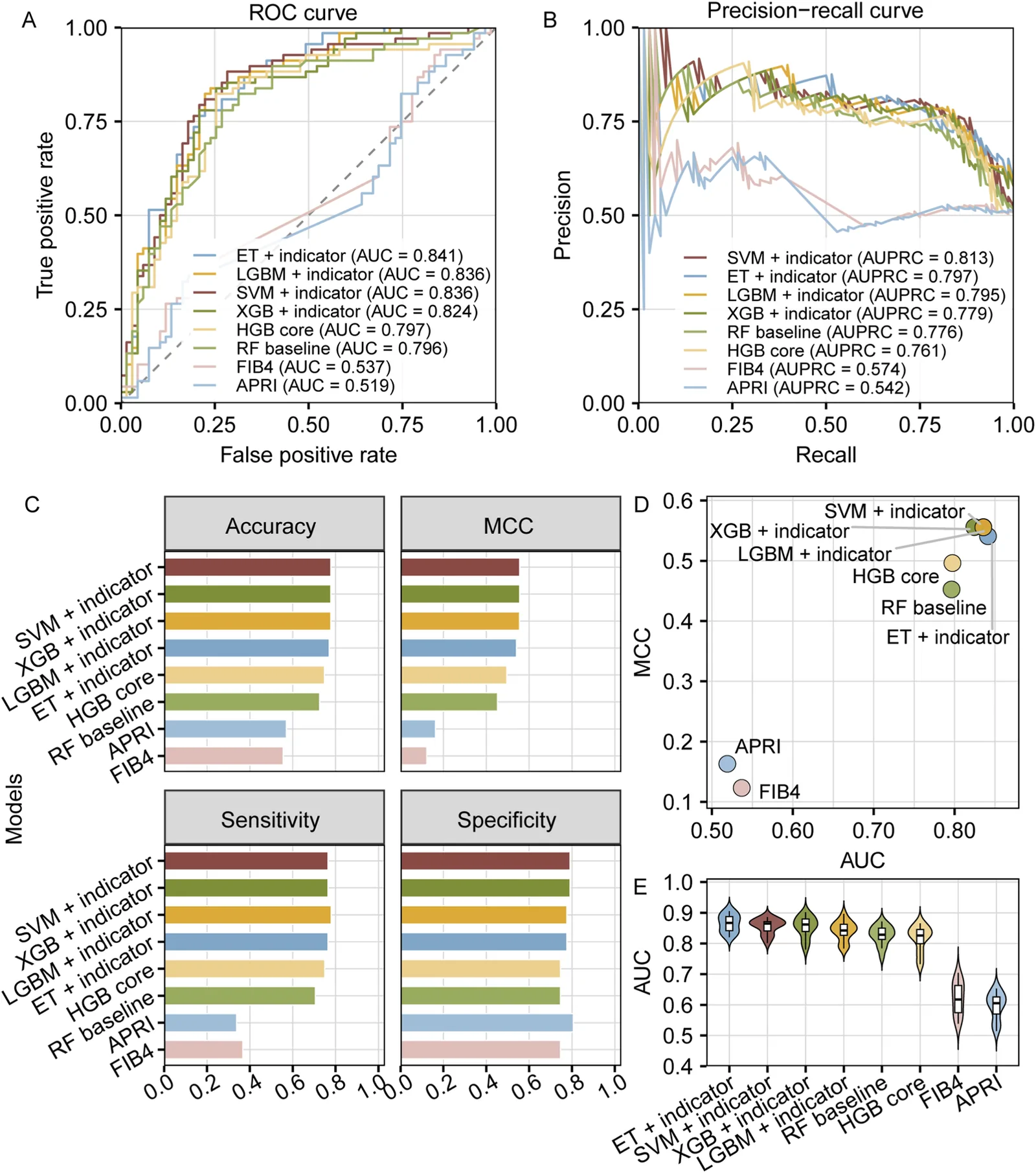
Comparative performance of clinical scores and machine-learning models for cirrhosis discrimination. **(A)** ROC curves and corresponding AUC values of different models in the independent testing set. **(B)** Precision–recall curves and corresponding AUPRC values of different models in the independent testing set. **(C)** Comparison of accuracy, MCC, sensitivity, and specificity across models. **(D)** Overall performance distribution based on testing-set AUC and MCC. **(E)** Violin plots showing cross-validation AUC distributions across models, used to assess model stability.

Classification metrics in the testing set showed similar overall performance for SVM + indicator, XGB + indicator, and LGBM + indicator, each with an accuracy of approximately 0.778 and MCC of approximately 0.556 (Figures 3C,D). ET + indicator achieved an accuracy of 0.770 and an MCC of 0.541. Although its accuracy and MCC were slightly lower than those of SVM + indicator, XGB + indicator and LGBM + indicator, ET + indicator had the highest AUC. For SN and SP, LGBM + indicator achieved the highest SN (0.779), whereas SVM + indicator and XGB + indicator had higher specificity, both approximately 0.791.

Cross-validation analysis showed that the AUC distributions of machine learning models were higher than those of FIB-4 and APRI (Figure 3E). ET + indicator, SVM + indicator, XGB + indicator, and LGBM + indicator constituted the group of highest-performing models (Figure 3E).

### 3.4 Missingness indicators improve discrimination in representative models

To evaluate whether missingness patterns contributed to model performance, models with and without missingness indicators were compared (Figure 4A). Incorporating missingness indicators increased testing-set AUC from 0.754 to 0.841 for ET, from 0.740 to 0.836 for SVM, and from 0.797 to 0.822 for HGB. These results indicated that explicit missingness indicators improved discrimination for all three representative models.

**FIGURE 4:**
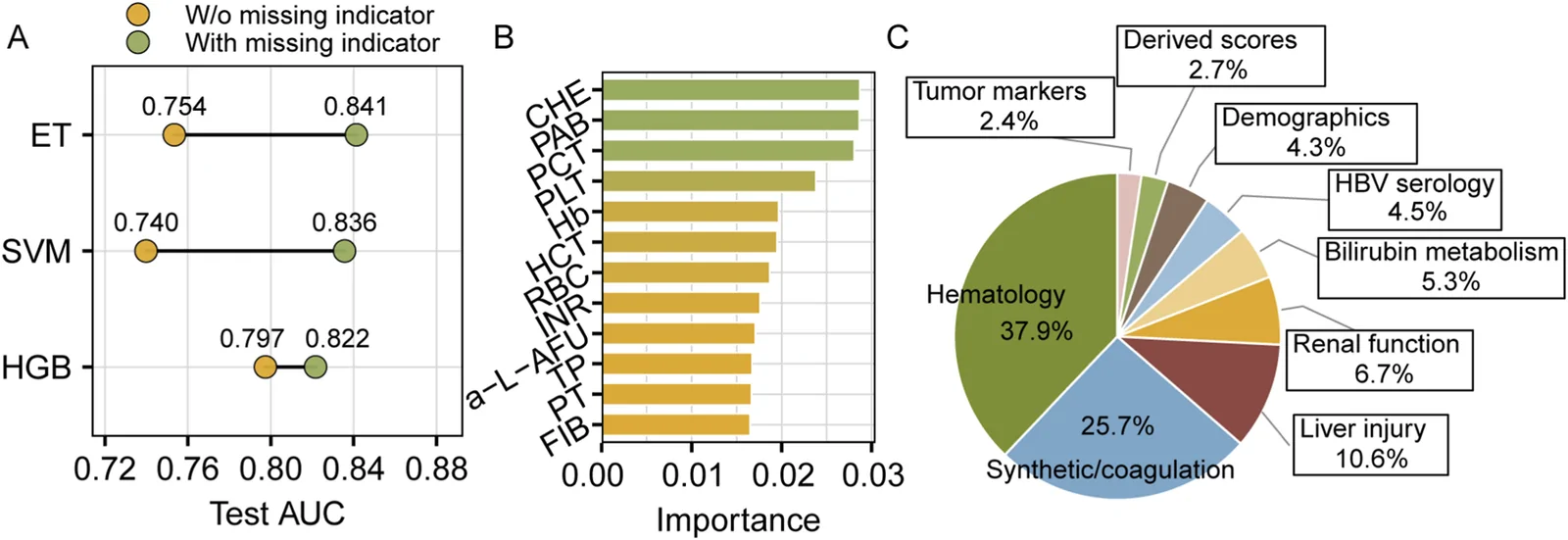
Incremental value of missing indicators and feature-level interpretation of model performance. **(A)** Comparison of testing-set AUC values for ET, SVM, and HGB models with and without missingness indicators. **(B)** Ranking of the most important features in the final model. **(C)** Proportional contribution of different feature categories to overall model importance.

Feature importance analysis identified measured variables and missingness indicators that contributed to model predictions (Figures 4B,C). Among measured variables, CHE, PAB, PCT, PLT, Hb, HCT, RBC, INR, α-L-AFU, TP, PT, and FIB contributed prominently. Several missingness indicators also contributed to model predictions, particularly the missingness status of AFP, HBeAg, and PIVKA-II. When aggregated by clinical category, feature importance was concentrated in haematological parameters (37.9%) and hepatic synthetic/coagulation markers (25.7%), followed by liver injury-related markers (10.6%), renal function indicators (6.7%) and bilirubin metabolism-related parameters (5.3%).

### 3.5 Threshold adjustment increased sensitivity for screening-oriented use

Using the ET + indicator model, we compared testing-set performance between the default classification threshold (0.50) and a screening-oriented threshold (0.42) (Figure 5). Lowering the threshold from 0.50 to 0.42 increased the number of true positives from 52 to 60 and reduced false negatives from 16 to 8. SN increased from 0.765 to 0.882, whereas SP decreased from 0.776 to 0.657, MCC increased slightly from 0.541 to 0.554. These results suggest that improved case detection was achieved without compromising overall classification quality (Figures 5A,B).

**FIGURE 5:**
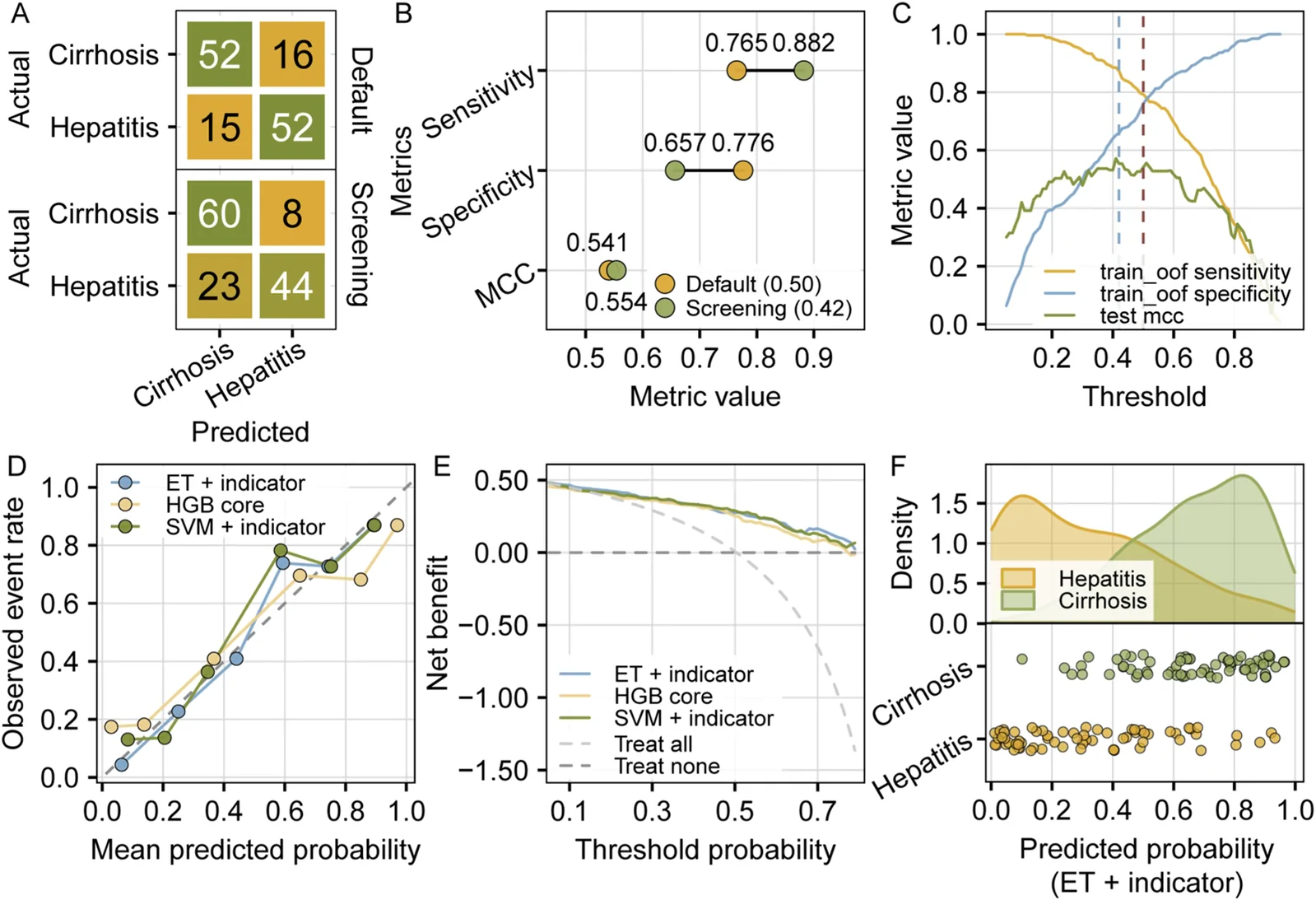
Threshold optimization, calibration and clinical utility of the ET + indicator model in a screening-oriented setting. **(A)** Confusion matrices in the independent testing set at the default threshold (0.50) and the screening-oriented threshold (0.42). **(B)** Comparison of sensitivity, specificity, and MCC between the two thresholds. **(C)** Threshold–performance curves based on out-of-fold predictions from the training set and results from the independent testing set. **(D)** Calibration curves for ET + indicator, HGB core, and SVM + indicator. **(E)** Decision curve analysis of representative models. **(F)** Predicted probability distributions of ET + indicator in the hepatitis and cirrhosis groups.

Threshold-curve analysis identified approximately 0.42 as a screening-oriented operating point with higher sensitivity and lower specificity than the default threshold (Figure 5C). Calibration analysis showed that the predicted probabilities of ET + indicator, HGB core, and SVM + indicator were broadly consistent with observed event rates, with ET + indicator showing closer agreement between predicted and observed probabilities across most probability intervals (Figure 5D). Decision curve analysis showed that all three models provided greater net benefit than the “treat all” and “treat none” strategies across the main range of threshold probabilities, with ET + indicator showing generally higher or comparable net benefit (Figure 5E). Predicted probability distributions showed that patients with cirrhosis tended to have higher predicted probabilities, whereas patients with hepatitis were more concentrated in lower probability ranges, with partial overlap between the two groups (Figure 5F). Overall, ET + indicator showed good discriminatory performance in the independent testing set, and threshold adjustment further enhanced its practical utility as a screening-oriented clinical tool.

## 4 Discussion

This study evaluated whether routinely collected laboratory variables could improve the distinction between hepatitis and cirrhosis in real-world clinical data. Machine learning models based on multivariable clinical features showed higher discriminatory performance than conventional scores, including FIB-4 and APRI. Models that explicitly incorporated missingness indicators generally performed better, and ET + indicator achieved the highest AUC in the independent testing set. After screening-oriented threshold optimization, ET + indicator also improved the detection of cirrhosis. Together, these findings suggest that missingness-aware modelling may add value to laboratory-based risk stratification for identifying cirrhosis among patients with hepatitis.

The observed group differences in clinical indicators were consistent with established features of cirrhosis. Patients with cirrhosis had lower CHE, PAB, PLT, and Hb, together with higher PT and TBIL, than patients with hepatitis. These differences correspond to impaired hepatic synthetic function, coagulation abnormalities, haematological alterations, and disturbed bilirubin metabolism (Abbas and Abbas, 2017; Li et al., 2023; Chen et al., 2022). Cirrhosis is therefore better represented as a multidimensional clinical phenotype than as an abnormality in a single marker. This provides a rationale for multivariable machine learning approaches, which can integrate laboratory signals across several pathophysiological domains rather than relying on the limited variables included in simplified clinical scores.

Feature importance analysis supported this interpretation. The dominant discriminatory signals were mainly derived from haematological parameters and markers of hepatic synthetic and coagulation function, consistent with the clinical pathophysiology of cirrhosis. These findings do not establish mechanistic relationships, but they indicate that the models used clinically interpretable laboratory domains when distinguishing cirrhosis from hepatitis.

A central methodological finding was that missingness patterns provide additional discriminatory information. Incorporating missingness indicators improved the performance of ET, SVM, and HGB models. This observation is consistent with the possibility that missingness in real-world clinical data is not completely random and may reflect clinical suspicion, disease severity, physician decision-making, healthcare accessibility, insurance or cost considerations, admission department, test-ordering pathways, or local institutional workflows. Therefore, missingness indicators should not be interpreted directly as intrinsic biological characteristics of patients or as disease biomarkers. Instead, they should be regarded as context-dependent signals embedded in the electronic medical record. Measured biological features remained the main contributors to prediction, and missingness indicators should be interpreted only as complementary information rather than as substitutes for laboratory measurements.

From a clinical perspective, ET + indicator may be more appropriate as a triage or screening-support model than as a definitive diagnostic tool. Lowering the classification threshold from 0.50 to 0.42 increased sensitivity from 0.765 to 0.882 and reduced false negatives, while maintaining similar overall classification quality. This trade-off is relevant to screening-oriented scenarios in which missed cirrhosis cases are a major concern. However, the model cannot replace abdominal imaging, liver stiffness measurement, liver biopsy, specialist assessment, or standard clinical diagnosis. Its appropriate role is to help identify patients who may require further evaluation rather than to confirm cirrhosis independently. Calibration and decision curve analyses further supported the potential value of the model for risk stratification, although prospective validation is required before clinical implementation.

Several limitations should be acknowledged. First, this was a single-centre retrospective study without external validation. The generalizability of the findings to other institutions, populations and clinical workflows therefore remains uncertain. Second, the benefit of missingness indicators may depend on institution-specific test-ordering practices, physician preferences, healthcare accessibility, and administrative workflows. Models that use missingness information may require recalibration or validation before being applied in settings with different clinical pathways. Third, participants and variables were pre-filtered, which may limit applicability to broader clinical populations or datasets with different missingness structures.

## 5 Conclusion

Machine-learning models based on routinely collected laboratory parameters improved discrimination between hepatitis and cirrhosis compared with conventional clinical scores in this real-world cohort. Incorporating missingness indicators added predictive information, suggesting that structured missingness patterns can support laboratory-based risk stratification when interpreted cautiously as institution-dependent clinical-process signals. The ET + indicator model may provide an auxiliary screening or triage-support tool for identifying patients who require further assessment, but it cannot replace imaging examinations, liver stiffness measurement, liver biopsy, specialist diagnosis, or other standard diagnostic procedures. Multicentre and prospective validation is required before clinical implementation.

## Data Availability

The data analyzed in this study is subject to the following licenses/restrictions: the data underlying this study were derived from electronic medical records of the Affiliated Hospital of North Sichuan Medical College. These data are not publicly available due to patient privacy, ethical restrictions, and institutional data-protection policies. De-identified data may be available from the corresponding authors upon reasonable request and with permission from the institutional ethics committee and the Affiliated Hospital of North Sichuan Medical College. Requests to access these datasets should be directed to yangqin@nsmc.edu.cn.
